# The Oura Ring Versus Medical‐Grade Sleep Studies: A Systematic Review and Meta‐Analysis

**DOI:** 10.1002/oto2.70181

**Published:** 2025-11-10

**Authors:** Sofia Khan, Alexa F. Ibrahim, Srivatsa Surya Vasudevan, Olivia E. Quatela, Douglas P. Nanu, Michele M. Carr

**Affiliations:** ^1^ Department of Otolaryngology, Jacobs School of Medicine and Biomedical Sciences University at Buffalo Buffalo New York USA; ^2^ Jacobs School of Medicine and Biomedical Sciences University at Buffalo Buffalo New York USA; ^3^ Department of Otolaryngology Louisiana State University Health Sciences Center Shreveport Shreveport Los Angeles USA; ^4^ Elson S. Floyd College of Medicine at Washington State University Spokane Washington USA

**Keywords:** actigraphy, diagnostic accuracy, meta‐analysis, Oura Ring, polysomnography, sleep monitoring, sleep tracking, wearable devices

## Abstract

**Objective:**

To evaluate the validity of the Oura Ring (OR; Oura Health Ltd.) in measuring sleep parameters compared to medical‐grade sleep studies including polysomnography (PSG) or actigraphy (ACT).

**Data Sources:**

PubMed, Scopus, and CINAHL.

**Review Methods:**

A systematic review and meta‐analysis was conducted in accordance with PRISMA guidelines. Studies were included if they evaluated sleep parameters measured simultaneously by the OR and PSG or ACT. Outcomes assessed included Total Sleep Time (TST), Sleep Efficiency (SE), Wake After Sleep Onset (WASO), Sleep Onset Latency (SOL), Light Sleep Time (LST), Deep Sleep Time (DST), and Rapid Eye Movement (REM) sleep time. Mean differences with 95% confidence intervals were calculated using a random‐effects model. A *P* < .05 was considered statistically significant.

**Results:**

Out of 2104 articles, 6 studies (n = 388) were included. There were no statistically significant differences between the OR and PSG/ACT for TST (MD: −2.97 min; 95% confidence interval [CI]: −10.27 to 4.33), SE (MD: −1.32%; 95% CI: −2.76 to 0.12), WASO (MD: 1.64 min; 95% CI: −12.57 to 15.86), SOL (MD: 0.48 min; 95% CI: −2.93 to 3.89), LST (MD: −4.27 min; 95% CI: −24.68 to 16.13), DST (MD: 1.39 min; 95% CI: −10.45 to 13.23), and REM sleep time (MD: −3.89 min; 95% CI: −17.23 to 9.46).

**Conclusion:**

The OR demonstrates comparable accuracy to PSG and ACT for commonly measured sleep parameters, supporting its utility as a self‐monitoring tool. This could prompt earlier clinical evaluation in symptomatic individuals or support remote monitoring of sleep.

Wearable devices that monitor real‐time health metrics have gained popularity in recent years, driven by consumer interest in health monitoring, preventive health, and fitness tracking. These devices offer individuals greater autonomy over their health. Among these devices, the Oura Ring (OR; Oura Health Ltd.) has gained significant traction due to its compact design and ability to track a range of physiologic parameters. It has increasingly been evaluated in research contexts as a self‐monitoring health tool tracking heart rate variability, body temperature, physical activity, and of our interest, sleep patterns.^1^, ^2^ The OR estimates sleep stages and efficiency using infrared photoplethysmography sensors, body temperature sensors, and an accelerometer. By analyzing patterns in heart rate variability, body temperature, and movement, the OR's proprietary algorithms classify sleep into light, deep, and REM stages and calculate metrics such as sleep onset latency and sleep efficiency.

Accurate assessment of sleep is crucial as certain sleep cycle patterns are linked to sleep disorders such as sleep apnea, narcolepsy, and insomnia, amongst others.^3^, ^4^, ^5^ Sleep quality and duration are tightly linked to cognitive function, metabolic health, and cardiovascular disease risk.^6^, ^7^, ^8^ Traditional methods such as polysomnography (PSG) or actigraphy (ACT) remain the medical‐grade standard for sleep evaluation, but are limited by their high cost and inconvenience. As a result, there has been growing interest in portable, consumer‐accessible devices that can reliably assess sleep outside of laboratory settings.

Validation studies have shown that while total sleep time estimates are highly accurate in the OR compared to PSG, stage classification has moderate accuracy.^2^, ^9^, ^10^ Given the growing use of wearable devices in personal health management, it is critical to assess their validity and reliability. The purpose of this systematic review and meta‐analysis is to evaluate the performance of the OR in measuring sleep parameters compared to gold‐standard methods, namely PSG and ACT, to determine its potential utility as a reliable self‐monitoring tool and its limitations within clinical application.

## Methods

### Search Criteria

**Figure 1:**
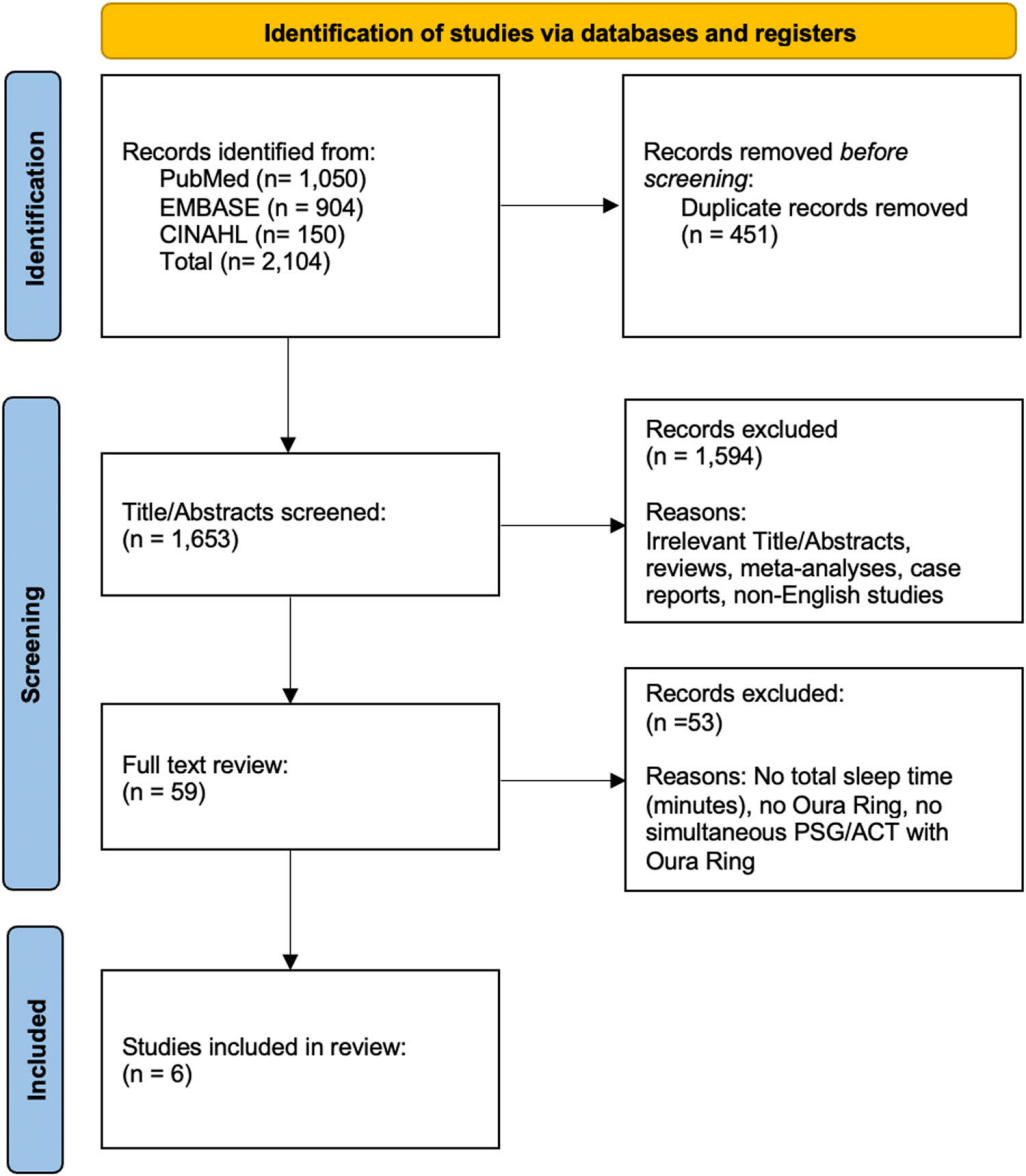
PRISMA flow diagram of the literature review. PRISMA, preferred reporting items for systematic reviews and meta‐analyses.

The study adhered to the Preferred Reporting Items for Systematic Reviews and Meta‐Analyses (PRISMA) guidelines (Figure 1).^11^ comprehensive literature search was performed across PubMed (National Library of Medicine, National Institutes of Health), Scopus (Elsevier), and CINAHL (EBSCO) from their inception through February 5, 2025. The PubMed search strategy incorporated both subject headings (eg, MeSH terms) and relevant keywords related to wearable devices and PSG or ACT. This strategy was adapted for Scopus and CINAHL by substituting MeSH terms with each database's specific subject headings. A detailed account of the search criteria and the number of results retrieved from each database is provided in Table 1. All references were imported into Covidence (Veritas Health Innovation Ltd.) for screening and review.

**Table 1:** Search Strategies and Results

Databases	Combination terms	Number of references obtained
PubMed	(sleep tracker OR wearable device OR ring OR watch) AND (Polysomnography OR actigraphy)	1050
EMBASE/SCOPUS	(“sleep tracker” OR “wearable device” OR “ring” OR “watch”) AND (“polysomnography” OR “actigraphy”)	904
EBSCO/CINAHL	(sleep tracker OR wearable device OR ring OR watch) AND (Polysomnography OR actigraphy)	150

### Selection Criteria

Studies involving patients who have undergone simultaneous PSG or ACT and sleep tracking with the OR were included. Studies were included if both medical‐grade sleep tracking devices and OR recorded TST at the minimum. Studies were excluded if sleep outcomes were not simultaneously measured by medical‐grade sleep trackers and the OR. Two reviewers (SK and AFI) first independently reviewed titles and abstracts to identify articles that met the inclusion criteria. Disagreements were resolved by way of discussion with the senior author (MMC). Both reviewers subsequently reviewed full texts independently to identify articles that met the inclusion criteria with disagreements after discussion with the senior author.

### Critical Appraisal and Quality Assessment

**Figure 2:**
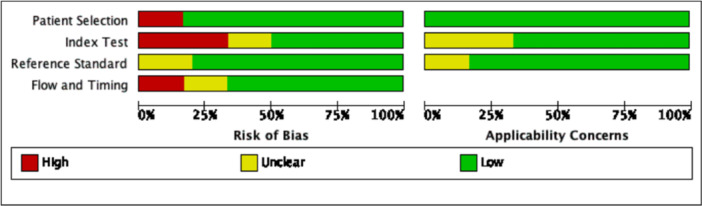
Risk of bias and applicability concerns graph: review authors' judgements about each domain presented as percentages across included studies.

Each study's level of evidence was assessed using the Oxford Centre for Evidence‐Based Medicine (OCEBM) criteria (Table 2).^18^ Risk of bias was assessed for each included study using the QUADAS‐C tool. In systematic reviews of diagnostic test accuracy, the QUADAS‐C tool is used to assess the risk of bias in comparative accuracy studies that evaluate 2 or more index tests. The QUADAS‐C tool evaluates four domains relevant to comparative diagnostic accuracy studies: patient selection, index test conduct and interpretation, reference standard conduct and interpretation, and flow and timing (Figure 2).^19^ For each domain, risk of bias was rated as “low,” “some concerns,” or “high.” An overall risk of bias judgment was assigned based on the highest level of risk identified across any domain. Applicability concerns were also considered but are reported separately. All assessments were conducted following the QUADAS‐C guidance criteria.

**Table 2:** Summary of Study Characteristics and Patient Demographics for Studies Included in the Systematic Review and Meta‐Analysis

Author	Sample size (n)	Country	LOE	Meanage, years (SD)	Sex (%)	Sleep study	Outcomes measured
Altini 2021^12^	118	Singapore, Finland, and USA	2	29.7 (16.7)	M: 44.9%, F: 55.1%	PSG	TST, LST, DST, REM
AsgariMehrabadi 2020^13^	45	Finland	3	33.1 (6.4)	M: 48.9%, F: 51.1%	ACT	TST, SE, WASO
deZambotti 2019^14^	41	USA	2	17.2 (2.4)	M: 68.3%, F: 31.7%	PSG	TST, LST, DST, REM, SOL, WASO
Kainec 2024^15^	53	USA	2	22.5 (3.5)	M: 41.5%, F: 58.5%	PSG	TST, LST, DST, REM, WASO
Robbins 2024^16^	35	USA	2	N/A	M: 42.9%, F: 57.1%	PSG	TST, LST, DST, REM, SE, SOL, WASO
Svensson 2024^17^	96	Japan	2	41.9 (13.8)	M: 49.0%, F: 51.0%	PSG	TST, LST, DST, REM, SE, SOL, WASO

Abbreviations: ACT, actigraphy; DST, deep sleep time; LOE, level of evidence; LST, light sleep time; N/A, no data; PSG, polysomnography; REM, rapid eye movement sleep time; SD, standard deviation; SE, sleep efficiency; SOL, sleep onset latency; TST, total sleep time; WASO, wake after sleep onset

### Data Extraction

Data were extracted independently by 2 reviewers and compared for accuracy. Disagreements were resolved by discussion. The Total Sleep Time (TST), Sleep Efficacy (SE), Wake After Sleep Onset (WASO), Sleep Onset Latency (SOL), Light Sleep, Deep Sleep, and REM Sleep were extracted from medical‐grade sleep trackers, either polysomnography (PSG) or ACT, and the OR.

### Statistical Analysis

All statistical analyses were performed using Comprehensive Meta‐Analysis (CMA) software, version 4. For studies comparing outcomes between OR and PSG, mean differences (MDs) and corresponding 95% confidence intervals (CIs) were calculated. Given the anticipated heterogeneity across studies, a random‐effects meta‐analysis model was employed. To assess the impact of a single study on the overall outcome, a sensitivity analysis was conducted using the leave‐one‐out method, whereby the meta‐analysis was repeated after sequentially removing each study. Publication bias was evaluated through visual inspection of funnel plots and Egger's regression test. All tests were 2‐sided, and a *P* < .05 was considered statistically significant.

## Results

### Search Results and Study Characteristics

The initial literature search yielded 2104 articles. Following duplicates removal, 1653 articles were included for screening. After title/abstract screening, 1594 records were excluded. Full‐text review screening of the remaining 59 articles was conducted, which yielded 6 final studies included in the meta‐analysis.^12^, ^13^, ^14^, ^15^, ^16^, ^17^ A PRISMA diagram detailing the literature search is shown in Figure 1. The included studies were published between 2019 and 2024, with the majority of the studies from the United States (50%). The Oxford Level of Evidence for each study was assessed and categorized as either Level 2 or 3, as detailed in Table 2. Quality assessment was performed using the QUADAS‐C tool for all 6 included studies (Figure 2).

### Patient Characteristics

In the quantitative analysis, 388 patients with a mean (SD) age of 64.7 (9.5) years were included to assess outcomes between OR and PSG. Outcomes measured include TST, SE, WASO, SOL, Light Sleep, Deep Sleep, and REM Sleep. The author, year, sample size, level of evidence, demographics (age and gender), and sleep outcomes were extracted for each included study (Table 2). All included studies evaluated generally healthy participants, with 5 of the 6 explicitly confirming the absence of sleep disorders through either self‐report, validated instruments, or polysomnography.

### Meta‐Analysis of OR Versus PSG

**Figure 3:**
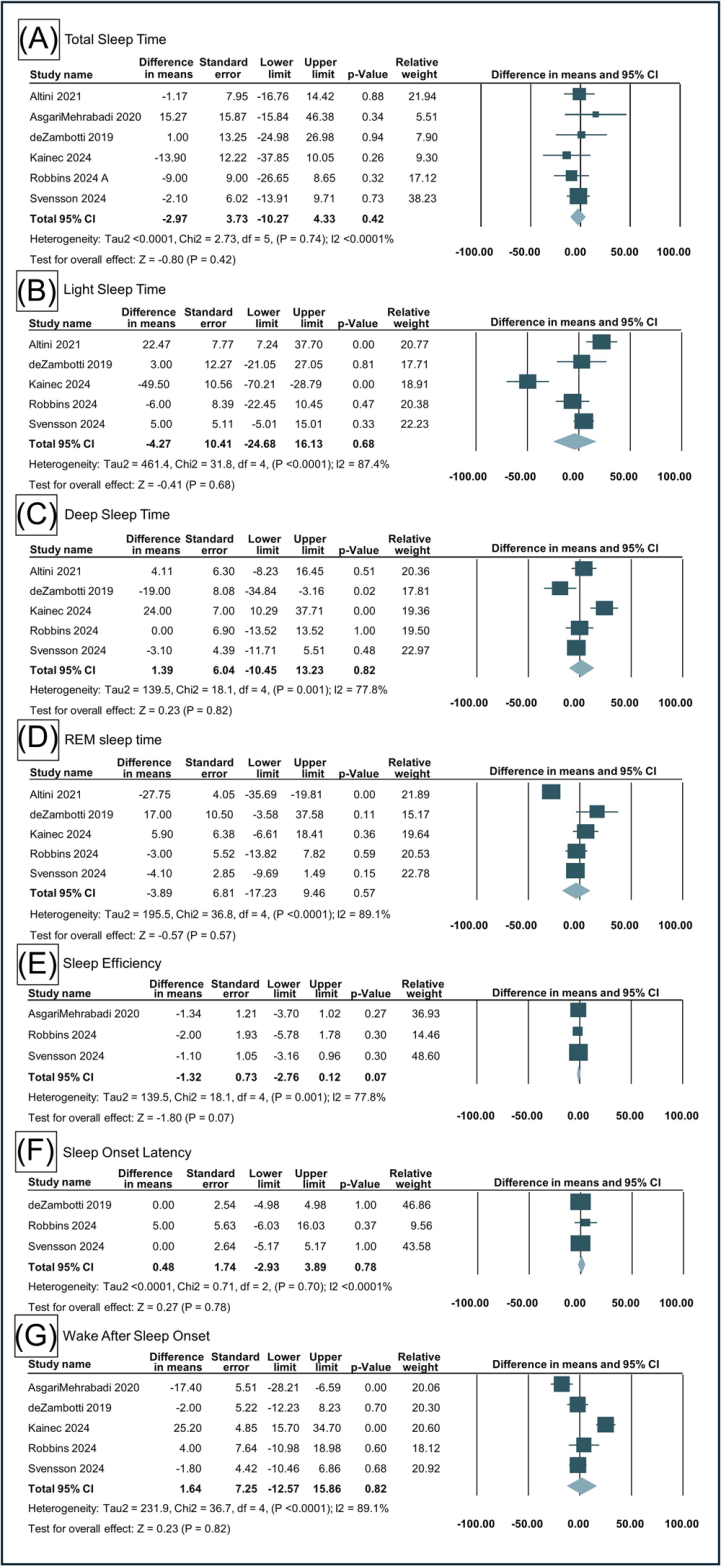
Forest plot illustration of sleep outcomes between OR and PSG/ACT. ACT, actigraphy; OR, Oura Ring; PSG, polysomnography.

Across 6 studies comparing the OR to PSG, there was no significant difference in TST, with a mean difference of −2.97 minutes (95% CI: −10.27 to 4.33; *P* = .42) (Figure 3). Analysis of LST showed a non‐significant mean difference of −4.27 minutes (95% CI: −24.68 to 16.13; *P* = .68). For DST, the OR measurements differed from PSG by an average of 1.39 minutes (95% CI: −10.45 to 13.23; *P* = .82), which was not statistically significant. SE time was slightly lower in the OR recordings compared to PSG, with a mean difference of −1.32% (95% CI: −2.76 to 0.12; *P* = .07), but this also did not reach significance (Figure 3). SOL differed by only 0.48 minutes between devices (95% CI: −2.93 to 3.89; *P* = .78), again showing no meaningful difference. WASO showed a small, non‐significant difference of 1.64 minutes (95% CI: −12.57 to 15.86; *P* = .82). Lastly, REM sleep time was slightly lower with OR compared to PSG, but the difference of −3.89 minutes (95% CI: −17.23 to 9.46; *P* = .57) was not statistically significant.

### Sensitivity Analysis

**Figure 4:**
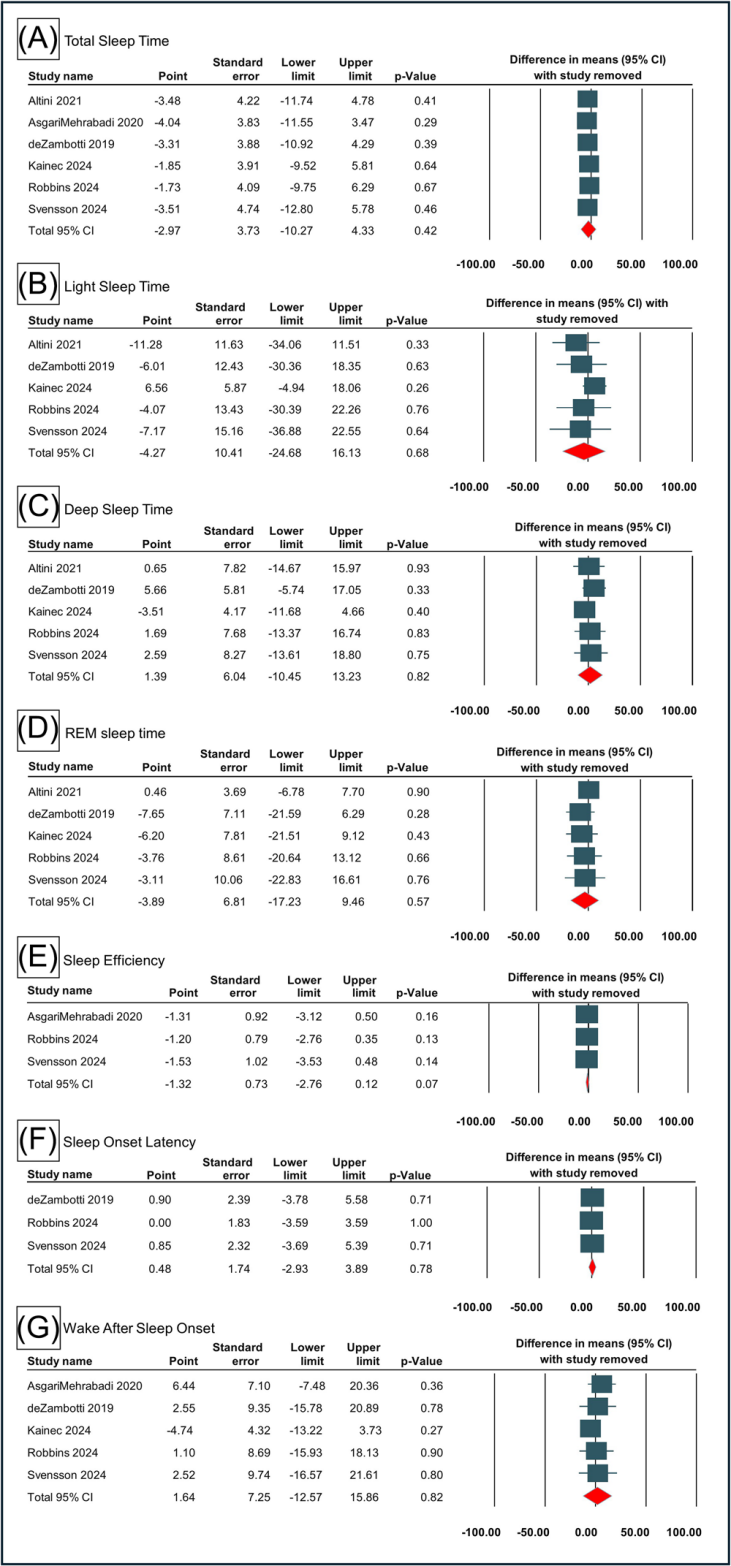
Sensitivity analysis with one‐study removal analysis for sleep outcomes between OR and PSG/ACT. ACT, actigraphy; CI, confidence interval; OR, Oura Ring; PSG, polysomnography.

On one‐study removal sensitivity analysis, there was no significant difference in the overall effects on TST, LST and DST outcomes as shown in Figure 4. For the SE, SOL and WASO, there were similar outcomes with no significant difference on sensitivity analysis (Figure 4).

### Publication Bias

**Figure 5:**
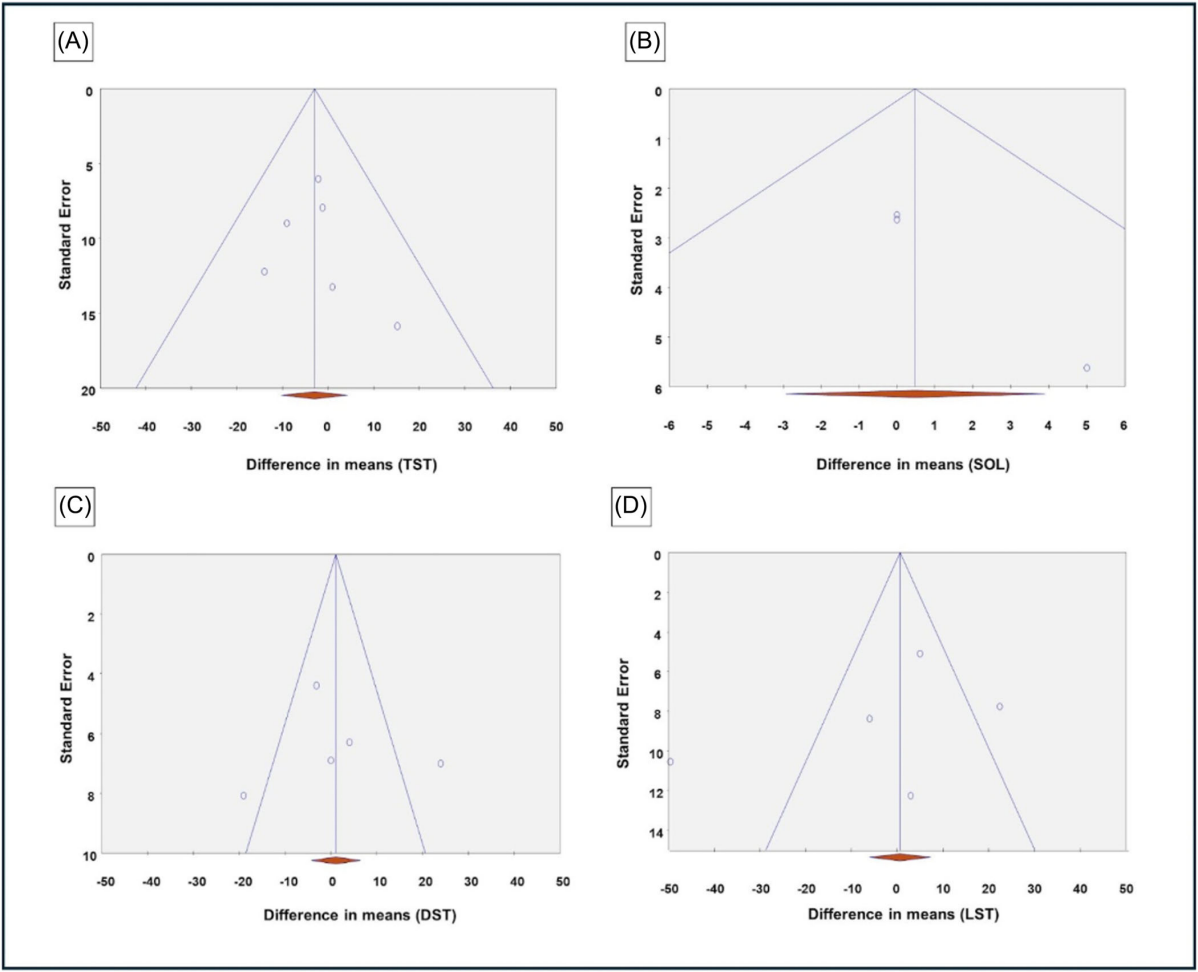
Funnel plot illustrating publication bias for (A) TST, (B) SOL, (C) DST, and (D) LST. DST, Deep Sleep Time; LST, Light Sleep Time; SOL, Sleep Onset Latency; TST, Total Sleep Time.

Egger's test for publication bias showed no significant bias for most sleep outcomes except for sleep onset latency (Figure 5), where a significant result was observed (*t* = 20, *P* = .03), suggesting potential publication bias for this outcome (Table 3).

**Table 3:** Publication Bias Assessment for OR Versus PSG Outcomes

Outcomes	Effect size (beta) with 95% CI	*t*‐value (Egger)	*P*‐value (Egger)	Publication bias
Total sleep time	0.42 (CI: −2.45‐3.29)	0.4	.71	Not significant
Light sleep time	−3.63 (CI: −17.3‐10.0)	0.84	.46	Not significant
Deep sleep time	0.74 (CI: −15.2‐16.6)	0.15	.89	Not significant
Sleep Efficiency	−1.02 (CI: −2.71‐0.67)	7.65	.08	Not significant
Sleep Onset Latency	1.64 (CI: 0.60‐2.68)	20	.03	Significant
Wake After Sleep Onset	−2.21 (CI: 31.4‐27.0)	0.24	.82	Not significant
REM sleep time	2.54 (CI: −9.40‐14.5)	0.68	.55	Not significant

Abbreviations: CI, confidence interval; OR, Oura Ring; PSG, polysomnography.

## Discussion

Sleep‐related complaints are among the most common reasons for referral to otolaryngology. Although polysomnography remains the diagnostic gold standard, its high cost, limited accessibility, and patient inconvenience often delay timely evaluation. Wearable devices offer a promising way to bridge these gaps, particularly in the current era of personalized medicine and digital health. Because sleep disturbances are strongly associated with cardiometabolic disease, mood disorders, and cognitive decline, accurate home‐based monitoring tools have the potential to play a critical role in both screening and longitudinal management of sleep disorders.^20^, ^21^, ^22^, ^23^

Findings from this meta‐analysis suggest that the Oura Ring, by demonstrating strong agreement with medical‐grade sleep studies across multiple parameters, may serve as a practical adjunct in ENT practice. Notably, no statistically significant differences were observed between the OR and PSG for TST, SE, SOL, WASO, or specific sleep stages. These findings are consistent with prior validation studies, which have shown that the OR has high sensitivity in detecting sleep versus wakefulness; however, our studies show high, rather than moderate, accuracy in distinguishing between sleep stages.^24^, ^25^, ^26^ Our sensitivity analyses further confirmed the robustness of these results, with no single study significantly altering the pooled outcomes. While Egger's test did not suggest publication bias for most outcomes, a significant result for sleep onset latency (*P* = .03) indicates potential selective reporting for this parameter. This should be considered when interpreting the agreement between the OR and PSG in measuring SOL.

While it is not a replacement for PSG in the diagnosis of conditions such as obstructive sleep apnea or narcolepsy, the OR can provide continuous data on sleep architecture and efficiency that may help flag deviations from normal patterns. This could aid in the screening and triage of patients presenting with nonspecific sleep complaints, prompting earlier clinical evaluation in symptomatic individuals. Given its accessibility and ease of use, the OR may therefore serve as a valuable screening or adjunctive tool in sleep medicine. The OR can also help in monitoring outcomes following surgical interventions such as adenotonsillectomy or nasal airway surgery, and support longitudinal tracking in patients. For instance, trends in reduced REM or deep sleep, elevated WASO, or decreased sleep efficiency over time may suggest underlying sleep pathology or worsening disease control.^27^, ^28^, ^29^ By providing a scalable, patient‐friendly, and accessible method for home‐based sleep monitoring, the OR may allow otolaryngologists to identify at‐risk individuals earlier, enhance patient engagement in their care, and prioritize formal testing for those most likely to benefit—all of which are particularly valuable in clinical settings.

Several limitations must be considered. First, heterogeneity in study designs, sample populations, and sleep metrics across included studies may influence the generalizability of the findings. Most included studies evaluated healthy adults, limiting extrapolation to populations with sleep disorders, comorbidities, or advanced age. Second, while the QUADAS‐C tool indicated a low to moderate risk of bias overall, variability in reference standards (PSG vs ACT) could introduce inconsistency. Finally, the evolving nature of wearable device algorithms necessitates continuous validation as technology advances.

Future research should focus on validating the OR and similar devices in more diverse populations, including individuals with sleep disorders such as obstructive sleep apnea and insomnia. Additionally, real‐world longitudinal studies assessing how wearable‐derived sleep metrics correlate with clinical outcomes would be valuable.

## Conclusion

The OR shows strong agreement with medical‐grade sleep studies across multiple sleep parameters, supporting its use as a reliable self‐monitoring tool. Its potential to identify abnormal sleep patterns highlights a promising role in the early detection and monitoring of sleep‐related disorders. This meta‐analysis shows that OR has comparable diagnostic accuracy with traditional sleep medical‐grade sleep studies. While the OR cannot yet replace clinical diagnostic testing, it offers a valuable, accessible adjunct in clinical ENT settings, research, and population health applications.

## Author Contributions

**Sofia Khan**, substantial contributions to the conception or design of the work and the acquisition, analysis, and interpretation of data for the work, drafting the work and reviewing it critically for important intellectual content, final approval of the version to be published and agreement to be accountable for all aspects of the work in ensuring that questions related to the accuracy or integrity of any part of the work are appropriately investigated and resolved; **Alexa F. Ibrahim**, substantial contributions to the conception or design of the work and the acquisition, analysis, and interpretation of data for the work, drafting the work and reviewing it critically for important intellectual content, final approval of the version to be published and agreement to be accountable for all aspects of the work in ensuring that questions related to the accuracy or integrity of any part of the work are appropriately investigated and resolved; **Srivatsa Surya Vasudevan**, substantial contributions to the conception or design of the work and the acquisition, analysis, and interpretation of data for the work, drafting the work and reviewing it critically for important intellectual content, final approval of the version to be published and agreement to be accountable for all aspects of the work in ensuring that questions related to the accuracy or integrity of any part of the work are appropriately investigated and resolved; **Olivia E. Quatela**, substantial contributions to the conception or design of the work and the acquisition, analysis, and interpretation of data for the work, drafting the work and reviewing it critically for important intellectual content, final approval of the version to be published and agreement to be accountable for all aspects of the work in ensuring that questions related to the accuracy or integrity of any part of the work are appropriately investigated and resolved; **Douglas P. Nanu**, substantial contributions to the conception or design of the work and the acquisition, analysis, and interpretation of data for the work, drafting the work and reviewing it critically for important intellectual content, final approval of the version to be published and agreement to be accountable for all aspects of the work in ensuring that questions related to the accuracy or integrity of any part of the work are appropriately investigated and resolved; **Michele M. Carr**, substantial contributions to the conception or design of the work and the acquisition, analysis, and interpretation of data for the work, drafting the work and reviewing it critically for important intellectual content, final approval of the version to be published and agreement to be accountable for all aspects of the work in ensuring that questions related to the accuracy or integrity of any part of the work are appropriately investigated and resolved.

## Disclosures

### Competing interests

None.

### Funding source

None.
